# Effect of urban environment on cardiovascular health: a feasibility pilot study using machine learning to predict heart rate variability in patients with heart failure

**DOI:** 10.1093/ehjdh/ztae050

**Published:** 2024-07-12

**Authors:** Valerie A A van Es, Ignace L J De Lathauwer, Richard G P Lopata, Astrid D A M Kemperman, Robert P van Dongen, Rutger W M Brouwers, Mathias Funk, Hareld M C Kemps

**Affiliations:** Department of Biomedical Engineering, Eindhoven University of Technology, 5600 MB Eindhoven, The Netherlands; Department of Built Environment, Eindhoven University of Technology, 5600 MB Eindhoven, The Netherlands; Department of Cardiology, Máxima Medical Centre, 5504 DB Veldhoven, The Netherlands; Department of Industrial Design, Eindhoven University of Technology, 5600 MB Eindhoven, The Netherlands; Department of Biomedical Engineering, Eindhoven University of Technology, 5600 MB Eindhoven, The Netherlands; Department of Built Environment, Eindhoven University of Technology, 5600 MB Eindhoven, The Netherlands; Department of Built Environment, Eindhoven University of Technology, 5600 MB Eindhoven, The Netherlands; Department of Industrial Design, Eindhoven University of Technology, 5600 MB Eindhoven, The Netherlands; Department of Industrial Design, Eindhoven University of Technology, 5600 MB Eindhoven, The Netherlands; Department of Cardiology, Máxima Medical Centre, 5504 DB Veldhoven, The Netherlands; Department of Industrial Design, Eindhoven University of Technology, 5600 MB Eindhoven, The Netherlands

**Keywords:** Machine learning (ML), Photoplethysmography (PPG), Heart rate variability (HRV), Congestive heart failure (CHF), Urban living environment

## Abstract

**Aims:**

Urbanization is related to non-communicable diseases such as congestive heart failure (CHF). Understanding the influence of diverse living environments on physiological variables such as heart rate variability (HRV) in patients with chronic cardiac disease may contribute to more effective lifestyle advice and telerehabilitation strategies. This study explores how machine learning (ML) models can predict HRV metrics, which measure autonomic nervous system responses to environmental attributes in uncontrolled real-world settings. The goal is to validate whether this approach can ascertain and quantify the connection between environmental attributes and cardiac autonomic response in patients with CHF.

**Methods and results:**

A total of 20 participants (10 healthy individuals and 10 patients with CHF) wore smartwatches for 3 weeks, recording activities, locations, and heart rate (HR). Environmental attributes were extracted from Google Street View images. Machine learning models were trained and tested on the data to predict HRV metrics. The models were evaluated using Spearman’s correlation, root mean square error, prediction intervals, and Bland–Altman analysis. Machine learning models predicted HRV metrics related to vagal activity well (*R* > 0.8 for HR; 0.8 > *R* > 0.5 for the root mean square of successive interbeat interval differences and the Poincaré plot standard deviation perpendicular to the line of identity; 0.5 > *R* > 0.4 for the high frequency power and the ratio of the absolute low- and high frequency power induced by environmental attributes. However, they struggled with metrics related to overall autonomic activity, due to the complex balance between sympathetic and parasympathetic modulation.

**Conclusion:**

This study highlights the potential of ML-based models to discern vagal dynamics influenced by living environments in healthy individuals and patients diagnosed with CHF. Ultimately, this strategy could offer rehabilitation and tailored lifestyle advice, leading to improved prognosis and enhanced overall patient well-being in CHF.

## Graphical Abstract

**Graphical Abstract:**
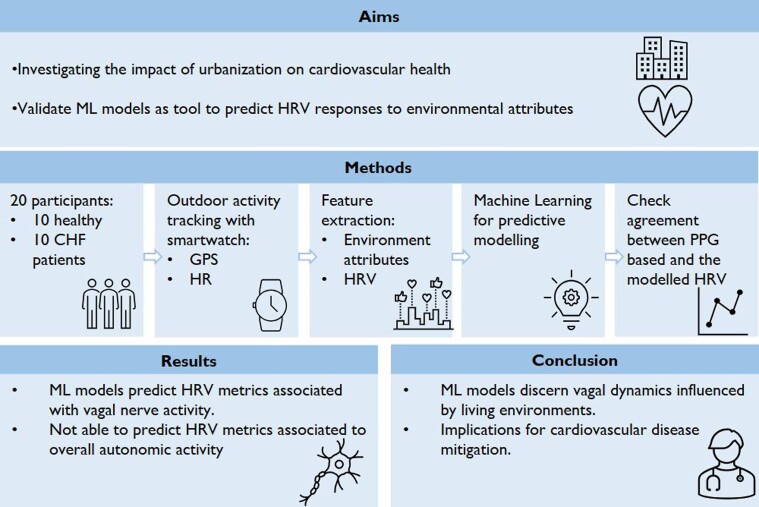

## Introduction

Congestive heart failure (CHF) is a major healthcare problem affecting millions of people worldwide.^1^ The number of patients with CHF keeps increasing due to improved survival after diagnosis and aging of the population.^2^ Congestive heart failure is a condition where the heart is no longer able to efficiently pump blood throughout the body. This inefficiency can result from either a diminished pump function or a reduced filling capacity of the heart. Congestive heart failure follows a chronic and progressive course, marked by acute episodes of deterioration.^3^ Congestive heart failure encompasses a wide array of symptoms, and it is associated with a diminished quality of life.^4,5^ Furthermore, CHF is intricately linked to an autonomic nervous system (ANS) imbalance, characterized by a sympathetic overdrive and decreased parasympathetic activity, which contributes to elevated mortality rates and significant socio-economic burdens.^6,7^ Currently, beta-blocker therapy stands as a firmly established cornerstone in the treatment of CHF, effectively mitigating sympathetic overdrive. However, alongside beta-blocker therapy, various studies have suggested that vagal nerve stimulation holds promise in addressing sympathetic overdrive. It is important to note that, as of now, vagal nerve stimulation therapy is primarily limited to experimental and research settings and is not yet applied in routine care.^6,8^

In recent years, a substantial global transition towards urban habitation has transpired. In 2008, a historic shift occurred as human society predominantly became urban for the first time. Looking ahead, the trend of global urbanization is expected to persist, with nearly 70% of the world’s population projected to be residing in cities by 2050.^9^ This shift has ushered a myriad of stressors, encompassing factors such as noise, air pollution, overcrowding, and the adoption of fast-paced lifestyles, culminating in adverse repercussions on both physical and psychological well-being.

While extensive research has emphasized the impact of environmental attributes on the ANS equilibrium in healthy subjects, there is a notable scarcity of studies investigating the influence of environments on the ANS in patients with CHF. It is now acknowledged that urban environments, characterized by densely populated areas and artificial structures, tend to activate the sympathetic branch of the ANS, while natural settings, such as green spaces or areas with abundant natural elements, elicit stimulation of the parasympathetic branch, facilitating stress recovery.^10–14^ Should these ANS responses exhibit consistency in patients with CHF, the therapeutic potential inherent in environmental settings could be effectively harnessed. For instance, the recommendation of telerehabilitation in an environment conducive to vagal nerve stimulation could be explored, thereby restoring ANS balance. In fact, research underscores the cardiovascular balance-restoring benefits of natural rehabilitation settings through vagal nerve stimulation in various populations.^12,15,16^ However, the application of this effect in real-world scenarios for patients with CHF has remained unexplored due to challenges in attributing ANS effects to environmental factors in uncontrolled settings. Nonetheless, advancements in wearable and tele-sensing technologies have rendered continuous monitoring of patients’ vital signs feasible. When integrated with artificial intelligence for data interpretation, this synergistic approach offers a quantifiable avenue for investigating the intricate relationship between environments and psychological responses.

Hence, the objective of this study was to evaluate the feasibility of employing machine learning (ML) models to predict ANS responses, as reflected in heart rate variability (HRV) metrics, during activities carried out in varied environmental contexts among both subjects diagnosed with CHF and healthy subjects. This assessment aims to validate the efficacy of integrating ML-based algorithms into existing wearables and, consequently, explore the potential relationship between specific environmental attributes and HRV responses.

## Methods

### Study design

**Figure 1:**
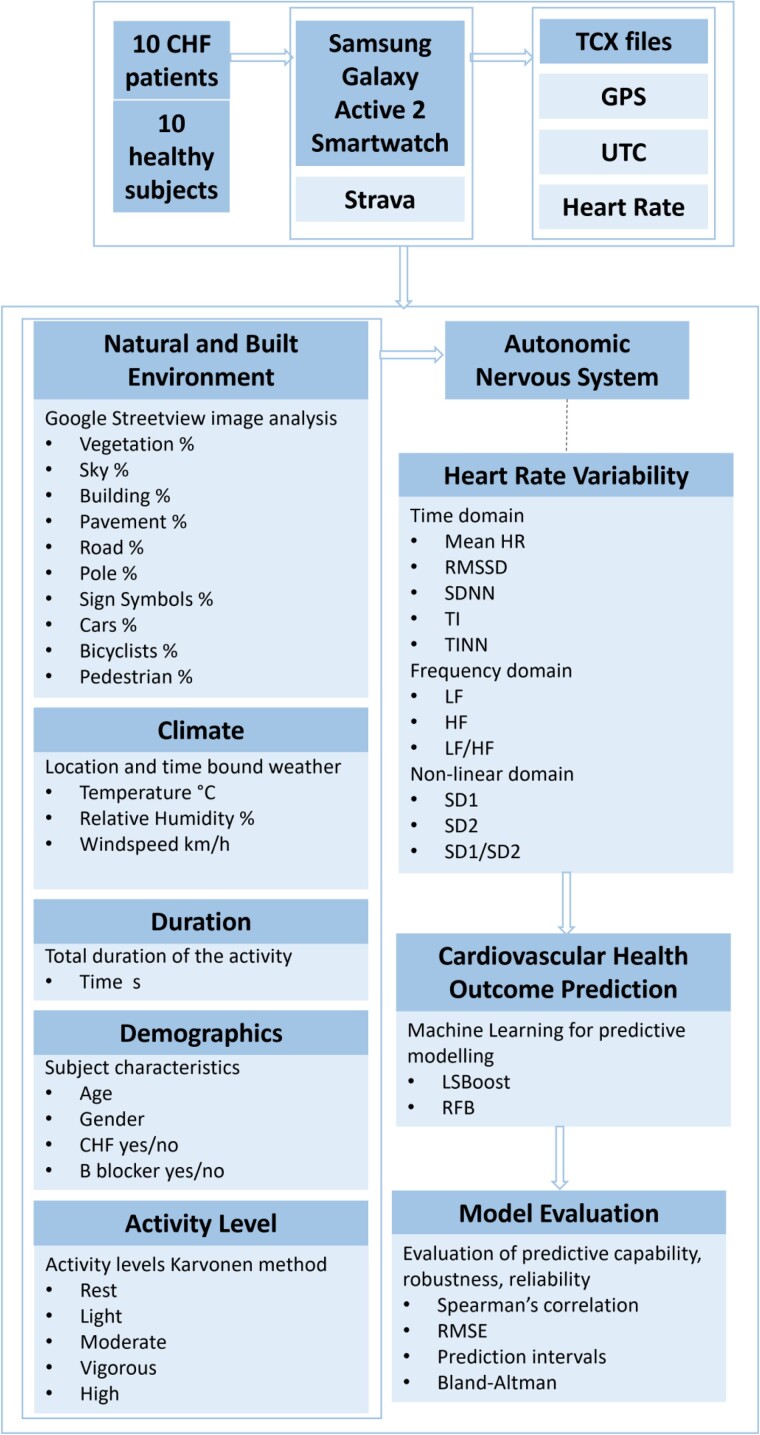
Research model of the study. The research model of the study illustrates the comprehensive flow of the entire investigation, commencing with data acquisition through the utilization of Samsung Galaxy Active 2 smartwatch-measured heart rate and GPS data, from which relevant features are extracted. These data then feed into the machine learning models that try to predict the impact they have on heart rate variability that reflects the autonomic nervous system. The machine learning models undergo thorough evaluation and analysis, to validate them as a tool to assess the relation in uncontrolled environmental settings and through subjects with various demographics.

This study employed a cross-sectional design to gather data on outdoor activity locations visited by the respondents remotely over a 3-week period, utilizing Samsung Galaxy Active 2 smartwatches. This duration allowed participants to capture their regular weekly activity patterns, providing sufficient time for adaptation to smartwatch measurements and minimizing potential measurement errors that might arise due to any issues with smartwatch usage. Participants were instructed to document a diverse array of activities in their outdoor living environment, encompassing but not limited to cycling, driving, walking, shopping, and other outdoor leisure or work-related activities, along with the concurrent recording of heart rate (HR) and global positioning system (GPS) data. The Strava application, installed on the smartwatches, facilitated the acquisition of these signals by accessing the photoplethysmography (PPG) sensor and GPS signal whenever a new type of activity was initiated. This enabled the collection of HR data over time at specific GPS coordinates. Apart from initiating and concluding an activity via the Strava app, participants were not required to furnish a comprehensive description of their activity. This information was subsequently extracted from the Strava recordings, minimizing the data collection burden as much as possible. To ensure compliance, participants were given the option to call if they had any doubts or uncertainties requiring clarification. To control the individual’s specific physical condition, the resting HR and maximum HR of the subject needed to be determined. The resting HR was obtained from the Samsung Galaxy Active 2 watch. For patients with CHF, a clinical physical exercise test, conducted by a specialist, was utilized to determine their maximum HR. In cases where the smartwatch recorded a maximum HR that exceeded the value determined during the clinical test, the smartwatch’s measurement was considered the true maximum HR. The resulting data were stored in Training Center XML (TCX) files, with 70% of the data allocated for training the ML models and 30% for validation. Further details on feature extraction for training the ML models will be expounded upon in section ‘Data analysis using ML models’. *Figure 1* provides a visual overview of the sequential stages undertaken in the study.

Ethical approval for the study was granted by the medical ethical committee of Máxima Medical Centre in Veldhoven, The Netherlands, with waiver reference number 2023-MMC-049. Additionally, approval was obtained from the Ethical Review Board of the Eindhoven University of Technology, The Netherlands, with reference number ERB2023BE26. Prior to participation, all individuals provided written informed consent in accordance with the principles outlined in the Declaration of Helsinki.

### Study population

Both healthy individuals and patients with CHF were recruited serving a dual purpose: firstly, to enhance our understanding of ANS responses triggered by environmental factors and secondly, to investigate whether these ANS responses exhibit similarity across individuals diagnosed with HF, characterized by sympathetic overdrive, and their healthy counterparts.^7^ To evaluate this presumed diversity in ANS responses between patients with CHF and healthy subjects, we utilized feature importance during the training of ML models. Notably, when the label indicating whether a subject was diagnosed with HF demonstrated high predictive power, it indicated a significant distinction in ANS responses induced by the environment between subjects diagnosed with HF and those in good health.

Healthy volunteers were recruited from the immediate social circles of the researchers, while patients with CHF were approached through the outpatient clinic. It is important to note that the subjects were not matched to the patients with CHF. Consequently, we had to meticulously label the collected HRV data with the demographics of the respective subjects to address the inherent heterogeneity between the subjects with CHF and healthy controls in the ML models. For the patients with CHF, the following inclusion and exclusion criteria were applied: age 18 years and older, diagnosed with symptomatic CHF of New York Heart Association Classes II–IV, regardless of the HF type or aetiology, possessing sufficient digital capacity, and being able to speak and read either Dutch or English. Subjects who met any of the following criteria were excluded from participating in the study: inability to comprehend the purpose and procedures of the study, limited mobility (e.g. due to orthopaedic limitations), lack of internet connection, untreated life-threatening cardiac arrhythmias, acute coronary syndrome within the past 3 months, uncontrolled hypertension, severe valvular disease, or scheduled (cardiac) surgery within the next 6 months.

### Data collection

Subjects collected HR time series simultaneously with the corresponding GPS coordinates while performing outdoor activities using the Strava application installed on Samsung Galaxy Active 2 smartwatches. Strava accessed the PPG sensor and GPS signal whenever a new type of activity was initiated. The resulting data were stored in TCX files, with 70% of the data allocated for training the ML models and 30% for validation.

**Figure 2:**
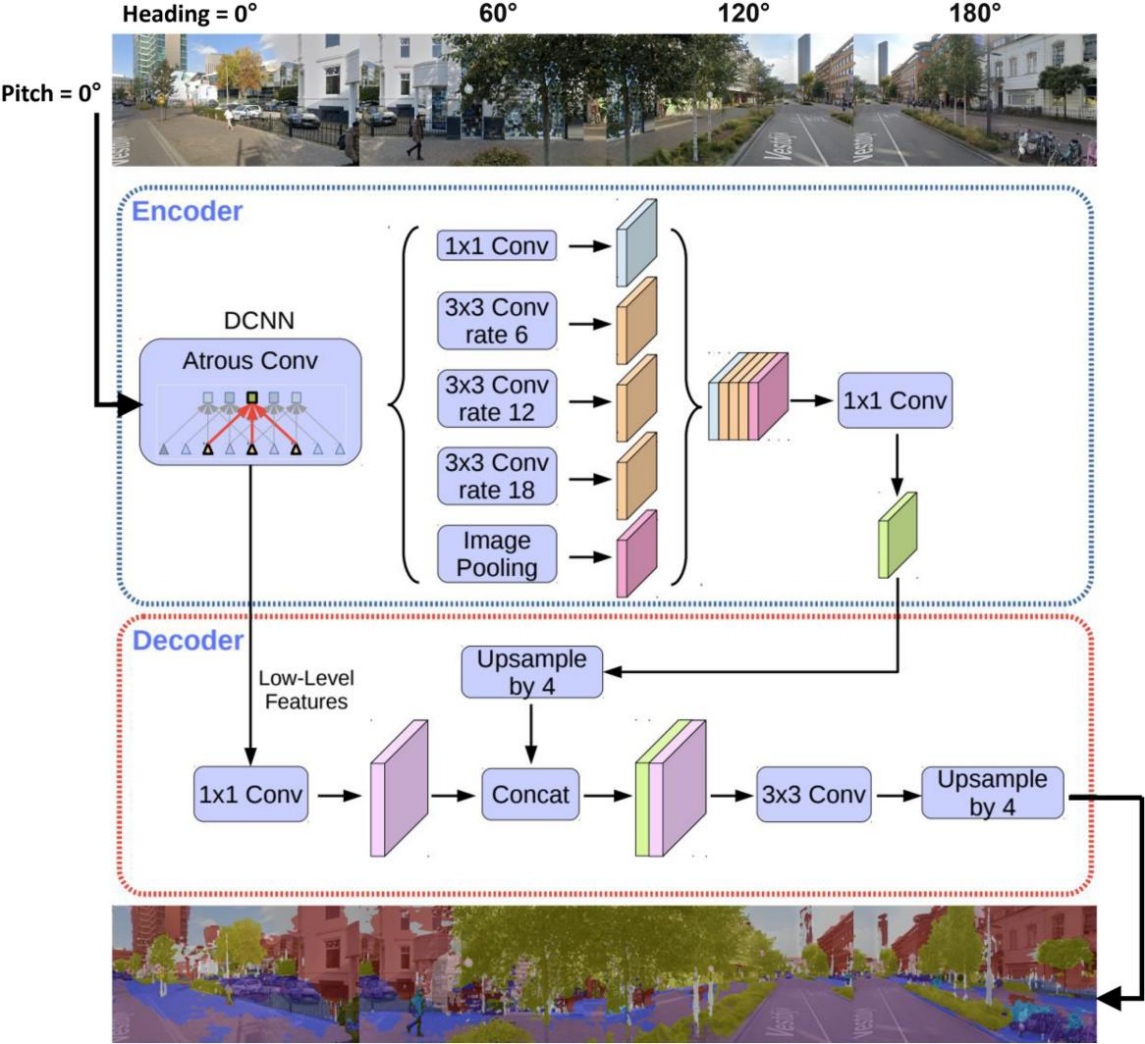
Semantic segmentation of the (urban) living environments. Segmentation of the 240° Google Street View images using the DeepLabV3+ model trained with Cambridge-driving Labeled Video data.

1. Predictor variables: environmental attributes
  To capture the visual landscape from the subject’s perspective at street level during an activity, we extracted four horizontal images every 10 min from Google Street View (GSV). These images covered the field of view (FOV) linked to the visual stimuli and were taken at the corresponding latitude (‘lat’) and longitude (‘lon’) coordinates GPS signal. The extraction process employed a 60° inter-image angle, with each image having a 60° FOV width. The camera was directed towards the subsequent lat and lon coordinates throughout the recorded activity, assuming that the subject was orienting their sight in the direction of movement. The camera settings are summarized in the application programming interface (API) parameters outlined in *Table 1*. It is important to acknowledge potential challenges in aligning these images with the actual visual experiences of the subjects, as variations in environmental context may occur due to factors such as seasonality or time of day. However, it is worth noting that all recorded activities took place during the summer and in daylight hours, thereby reducing variability in participants’ visual experiences.
  Deep learning techniques were utilized for semantic image segmentation to quantify visual stimuli. This involved identifying elements such as plants, buildings, sky, and roads within the GSV images. The percentage of each element present was calculated to quantify the environmental context in which the subject was engaged in an activity. This process is depicted in the ‘Natural and Built Environment’ box as shown in *Figure 1*.^17,18^ For this purpose, a pre-trained DeepLabV3+ network was utilized, having been trained on the Cambridge-driving Labeled Video Database (CamVid), which encompasses ten scenery classification groups (vegetation, sky, building, pavement, road, pole, signs symbols, cars, bicyclists, and pedestrians) as depicted in *Figure 2*. The Scenery View Index (SVI), a modification of the Green View Index, was subsequently employed to calculate the proportion of pixels within each classification group.^17,19^ This calculation, considering the API parameters outlined in *Table 1*, was carried out as follows:
  $$\mathrm{SVI}=\frac{\sum_{\;j=1}^{n}\;\sum_{i=1}^{m}\;A_{g,j}}{\sum_{\;j=1}^{n}\;\sum_{i=1}^{m}\;A_{t}{,}_{j}}\times 100\text{\%}$$
  where *A_gj_* represents the number of pixels belonging to one of the 10 classes, represented by *g*, extracted by the semantic segmentation algorithm in the horizontal direction *i* and vertical direction *j*. Similarly, *A_tj_* represents the total area of the GSV obtained image pixels in the horizontal direction *i* and the vertical direction *j*. The parameters *m* and *n* represent the numbers of horizontal and vertical directions, respectively. For the SVI calculation in this study, the value of *m* was set to 4 to achieve a 240° view, with an inter-picture angle of 60°, while *n* was set to 1, representing a pitch angle of 0°. Subsequently, the percentage of pixel assigned to each class during a location-bound activity served as the predictive input for the ML models. Concerns may arise regarding discrepancies in the percentage of vegetation and sky compared with metadata obtained from GSV. However, the deep learning algorithm effectively segments vegetation, even in scenarios such as trees without leaves during winter, still categorizing them as vegetation. Consequently, it maintains consistency in the percentage of pixels assigned to the vegetation class. Similarly, pixels designated as sky, including those with clouds on rainy days vs. clear skies, are equally distributed to the ‘sky’ class.
2. Target variables: HRV parameters
  The HRV parameters outlined in *Table 2* were obtained by extracting the inter-beat interval (IBI) series from the PPG signals, estimating the time intervals between successive systolic peaks.^20,21^ The automatic beat correction algorithm within the Kubios HRV tool was utilized to correct beats. Artefacts were identified from the differences between successive IBI series (dIBIs) series, which comprises differences between successive IBI intervals estimated from the PPG signal. This series offers a reliable method to distinguish ectopic and misplaced beats from the normal sinus rhythm. A time-varying threshold was employed to separate ectopic and normal beats. To accommodate varying HRV levels, this threshold was estimated from the time-varying distribution of the dIBI series. Following correction, this yielded a series of normal-to-normal (NN) intervals.^22^ The duration of the NN series for calculating HRV metrics will be determined by the shortest activity duration lasting longer than 10 min, regardless of its intensity type or environmental context, among all recorded activities. Each IBI corresponds to a cardiac cycle, allowing for the computation of HR as the inverse of the IBI duration. The average HR signifies the mean HR value over the signal duration. Beyond assessing average HR, various features were extracted from HRV across time, frequency, and non-linear domains. The HRV parameters under analysis were systematically divided into two distinct groups, each showcasing its ability to depict either vagal tone or overall autonomic activity, without specific physiological correlates regarding sympathetic and parasympathetic modulation at the sinus node. The first group, capable of forecasting vagal modulation, comprises HR, root mean square of successive interbeat interval differences (RMSSD), high frequency power (HF), low- and high frequency power (LF/HF), and Poincaré plot standard deviation perpendicular to the line of identity (SD1), while the second group offers a more general description of overall autonomic cardiac activity, including the standard deviation of NN intervals (SDNN), the integral of NN interval histogram divided by its height (TI), baseline width of the NN interval histogram (TINN), low frequency power (LF), the Poincaré plot standard deviation along the line of identity (SD2), and SD1/SD2.^23–25^ This classification played a crucial role in elucidating the ML model’s predictive capability regarding parameters and their implications in terms of autonomic cardiac regulation. It is important to note that these groups were established for later-stage result interpretation, with HRV metrics remaining unlabelled during the training and testing phases of the ML models.
3. Control variables: climate, activity duration, demographics, and activity level
  Control variables were incorporated, which included climate, activity duration, demographics, and activity level. This approach enabled the isolation of the variables of interest, specifically examining how the predictor variables, representing environmental attributes, influence the target variables, namely HRV metrics. This incorporation is justified by previous studies that have established statistically significant associations between these features and HRV.^23,24,26–28^ For each recorded activity, local weather conditions were extracted using GPS coordinates through the Open-Meteo API, encompassing features such as temperature, relative humidity, and wind speed. The duration of each activity was contingent upon the start and stop times as initiated by the participant. Given the uncontrolled nature of the settings in which activities were conducted, exposure duration to environmental contexts could vary, potentially influencing HRV responses, necessitating correction. Demographic data included age, gender, CHF presence, and usage of beta blockers. Activity levels were classified using the Karvonen method, accounting for each subject’s fitness level based on individual resting and maximum HRs. HR reserve (HRR) was calculated to categorize activities into intensity levels, distributed as follows: rest (<20% of HRR), light (20–39% of HRR), moderate (40–59% of HRR), vigorous (60–84% of HRR), and high (85–100% of HRR).^29,30^

While we aimed to include variables widely recognized in the literature, we acknowledge the possibility of other unaccounted factors influencing HRV. Due to the complexity and multifaceted nature of HRV determinants, this study focuses on control variables that are assumed to result in significant changes in HRV metrics within the medium measurement duration range (5 min < activity time < 24 h). This approach is chosen to strike a balance between capturing relevant influences and avoiding overfitting the ML models with an exhaustive set of variables. Future research may explore additional factors, acknowledging the evolving landscape of HRV research.

**Table 1:** API parameters for Google Street View extraction

Parameter	Description	Example
Size	Output size in pixels	Size = 600 × 600
Location	Lat and lon coordinates	Lat = 51.4381 Lon = 5.4752
FOV	Horizontal FOV	FOV = 60°
Inter-picture angle	Angle between subsequent images	Inter-picture angle = 60°
Heading	Compass heading of camera	Heading = 0° Heading = 60° Heading = 120° Heading = 180°
Pitch	Up or down angle of camera	Pitch = 0°
Key	Developer’s key	Key = ‘fill in your API key’

**Table 2:** Heart rate variability metrics in the time, frequency, and non-linear domains

HRV metrics	Unit	Description
*Time domain*		
HR	/min	Average HR. Reflects vagal activity
RMSSD	ms	Root mean square of successive inter beat intervals (IBIs) differences. Reflects vagal activity
SDNN	ms	Standard deviation of NN intervals. Reflects overall autonomic activity
TI	—	Integral of NN interval histogram divided by its height. Reflects overall autonomic activity
TINN	ms	Baseline width of the NN interval histogram. Reflects overall autonomic activity
*Frequency domain*		
LF	ms^2^	Absolute power of the low-frequency power (0.04–0.15 Hz). Reflects overall autonomic activity
HF	ms^2^	High frequency power (0.15–0.04 Hz). Reflects vagal activity
LF/HF	—	Ratio of LF-to-HF absolute power. Reflects vagal activity
*Non-linear domain*		
SD1	ms	Poincaré plot standard deviation perpendicular to the line of identity. Reflects vagal activity
SD2	ms	Poincaré plot standard deviation along the line of identity. Reflects overall autonomic activity
SD1/SD2	—	Ratio of SD1-to-SD2 standard deviation. Reflects overall autonomic activity

### Data analysis using ML models

Data analysis using ML models offers a novel approach to examine and predict HRV responses in both patients with CHF and healthy individuals during activities conducted in various environments characterized by diverse environmental attributes. Integrating ML-based data analysis and predictive models into current remote monitoring systems can provide valuable insights for physicians prescribing rehabilitation plans. This section explains how different models are selected and trained to predict HRV responses associated with activities performed in different environments across subjects diagnosed with CHF and their healthy counterparts. All processing steps were executed using MATLAB (R2020A, MathWorks Inc., Natick, MA, USA).

1. Model selection
  Prior to model training, the normal distribution of input features underwent assessment via the Shapiro–Wilk test.^31^ This examination encompassed both predictive and control features, disclosing noteworthy deviations from normality (*P* < 0.05) across all features. Consequently, this study elected to utilize predictive models able to effectively handle such non-normal data characteristics. Specifically, least-squares gradient boosting (LSBoost)^32^ and Random Forest Bagging (RFB)^33^ were chosen for their suitability in accommodating non-normal data distributions.
2. Model training
  The training phase primarily centred on identifying optimal hyperparameters, presenting a challenging task in configuring parameters for data-driven modelling with the most suitable ML model. To streamline this intricate process, we adopted a Bayesian strategy.^34^ The overarching aim was to establish an ensemble regression model with the minimal estimated leave-one-out cross-validation loss.^35^ The hyperparameter search space encompassed variables such as the number of learning cycles, the learning rate for the LSBoost model, and the minimum leaf size for the tree learner. Ultimately, the model characterized by the set of hyperparameters that maximized the desired performance metric, as determined through five-fold cross-validation, was selected to predict the HRV parameter induced by the activity environment. It should be noted that hyperparameter optimization was conducted on 70% of the data allocated for model training.
3. Model evaluation
  The overall performance of the trained models, encompassing their predictive accuracy and reliability, underwent evaluation through a series of quantitative metrics. Initially, the acquired data underwent partitioning into training and test sets. The latter set served the purpose of comparing the predicted outcomes of the model with the actual values of the HRV parameters derived from the PPG signal. To gauge the predictive accuracy and reliability of the ML models, a suite of statistical analyses was conducted post-examining the residuals (*y*_pred_ − *y*_tar_) for their conformity to a normal distribution (Shapiro–Wilk’s test yielded *P* > 0.05 for all residuals, indicating normal distribution). The applied statistical analyses encompassed Spearman’s correlation analysis,^36^ calculation of the root mean square error,^37^ prediction intervals for non-simultaneous observations with 95% confidence interval,^38^ and Bland–Altman analysis including the mean bias and its 95% confidence intervals (expressed by mean differences ± 1.96*σ*).^39^ All statistical analyses were executed using MATLAB (R2020A, MathWorks Inc., Natick, MA, USA), with a significance level set at *P* < 0.05 for each analysis.

## Results

### Patient characteristics

A cohort of 20 subjects was enrolled, encompassing 10 healthy individuals and 10 patients diagnosed with CHF. Notably, one patient with CHF withdrew from the study due to technical issues with the smartwatch. Among the healthy individuals, there were four males and six females, with ages ranging from 23 to 65 years. Within the patient group, there were 10 males, ages ranging from 48 to 85. Details regarding the patient characteristics are presented in *Table 3*.

**Table 3:** Patient characteristics (*n* = 10)

Age (years)	76 (64–81)
Gender	
Male	10
Female	0
HF type	
HfrEF	5
HfmrEF	2
HFpEF	3
LVEF (%)	39 (36–52)
NYHA class	
NYHA II	9
NYHA III	1
HF aetiology	
Ischaemic	4
Non-ischaemic	6
Medication	
Beta-blocker	10
ACE-I or ARB	4
Sacubitril /valsartan	4
Sodium/glucose cotransporter 2 inhibitor	9
MRA	5
Diuretics	6

Values are presented as numbers or median (interquartile range).

### Data characteristics

**Figure 3:**
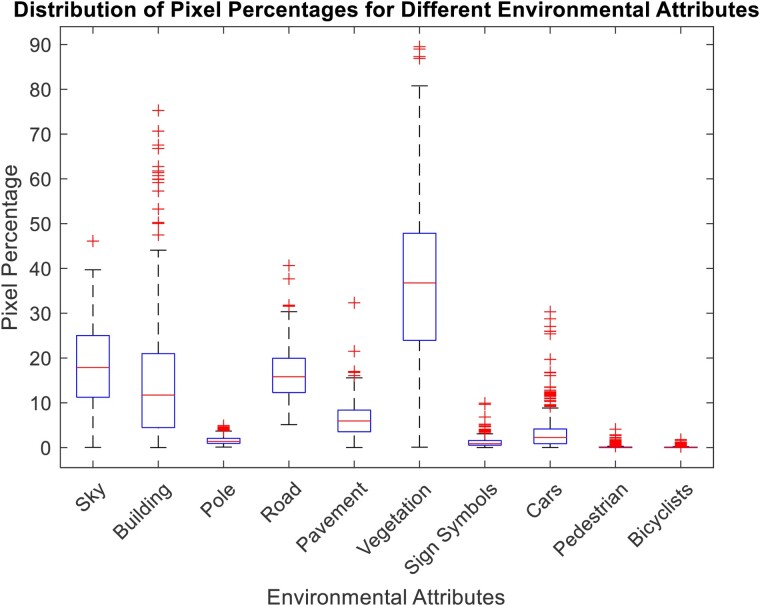
Box plots depicting the distribution of pixels attributed to environmental features as recorded by the participants.

The dataset comprised 367 sets of data collected by participants. From this dataset, 70% (257 samples) were utilized for training the models, while the remaining 30% (110 samples) were reserved for validating model performance. These data collections spanned various environmental contexts, with the pixel distribution segmented from the GSV images, as depicted in *Figure 3*. The participants’ recorded activities encompassed environments rich in vegetation. Additionally, they captured activities within built environments, characterized by the accumulation of pixels associated with categories such as ‘building’, ‘pole’, ‘road’, ‘pavement’, ‘sign symbols’, and ‘cars’. The recorded activities ranged from 15 to 30 min. To ensure the continuous extraction of HRV metrics, the NN intervals were clipped to 15-min intervals, from which the HRV metrics were subsequently calculated.

### Feature importance

**Figure 4:**
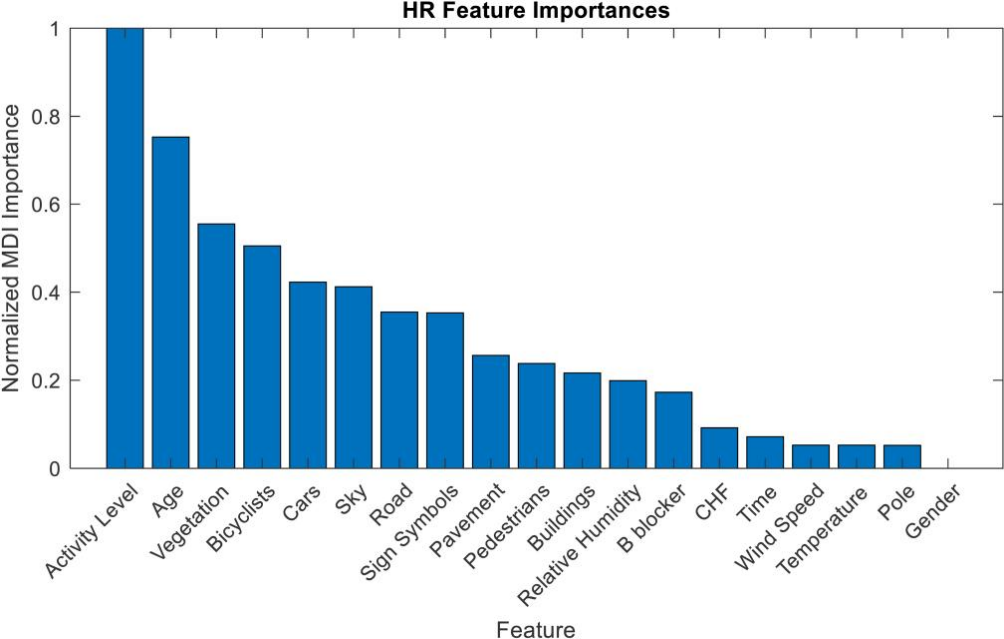
Mean decrease in impurity feature importance for the machine learning model forecasting the heart rate metric. A higher mean decrease in impurity value indicates a greater significance of the feature in predicting the target variable.

Mean decrease in impurity (MDI) feature importance has been calculated for all input variables, indicating the average reduction in impurity across decision trees when a feature is used to split nodes. A higher MDI value signifies greater importance in predicting the target variable, such as HRV metrics. *Figure 4* illustrates how environmental attributes enhance prediction purity for HR responses, with activity level and age being the most influential factors. Vegetation presence notably reduces impurity in predictions. Gender differences and CHF diagnosis have less impact on HR response predictions than expected.

### ML models

For each HRV metric, the best ML model was selected using Bayesian model evaluation with leave-one-out cross-validation.^35^ The model choice was based on the improvement in predictive power by using either LSBoost or RFB, along with the optimal number of learning cycles and the minimum leaf size. Each parameter had its own model as represented in *Table 4*.

**Table 4:** Best estimated feasible point and its machine learning model settings

HRV metrics	Method	Learning cycles	Min leaf size
*Time domain*			
HR	LSBoost	38	1
RMSSD	RFB	10	1
SDNN	LSBoost	106	69
TI	LSBoost	146	8
TINN	RFB	344	4
*Frequency domain*			
LF	RFB	48	4
HF	LSBoost	76	49
LF/HF	RFB	108	15
*Non-linear domain*			
SD1	RFB	10	8
SD2	RFB	496	4
SD1/SD2	LSBoost	15	87

**Figure 5:**
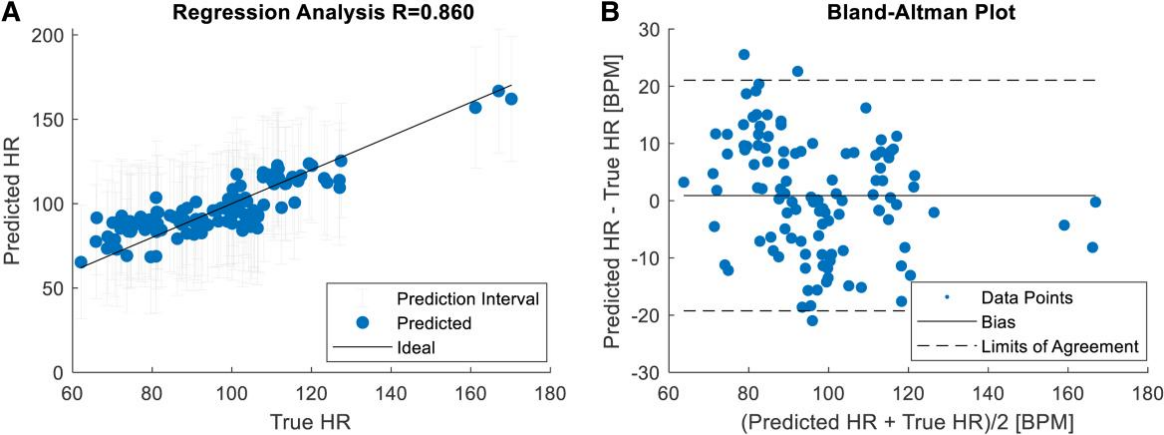
Graphical representation of the machine learning model predicting heart rate. (*A*) A regression plot illustrates the heart rate values obtained from the machine learning predictions alongside the absolute measurements, accompanied by prediction intervals for non-simultaneous observations at a 95% confidence level. (*B*) A Bland–Altman plot displays the heart rate values obtained from the machine learning predictions compared with the absolute measurements.

The findings from our analyses, detailed in *Table 5*, revealed robust model predictions for HRV metrics associated with the vagal nerve, such as HR, RMSSD, HF, LF/HF, and SD1. *Figure 5A* and *B* provides a visual representation of the Spearman’s correlation analysis and the Bland–Altman analysis of ML model’s HR predictions, respectively. Initial results show promise. However, it is crucial to acknowledge the ±20 b.p.m. margin of error in HR predictions, suggesting the need for further validation and refinement, as current margins of error are insufficient for monitoring conditions related to vagal activation, potentially leading to misinterpretation or mismanagement.

**Table 5:** Findings from the statistical analysis indicating the collective efficacy of the trained models. a) Regression analysis: RMSE and Spearman’s correlation coefficient *R* with statistical significance (*P* < 0.05). The highlighted HRV values in bold are those exhibiting a moderate-to-strong monotonic relationship (0.44 < *R* < 0.86) correlation (*R* > 0.4). b) Prediction intervals (95% confidence level): mean error and its standard deviation (*σ*). The HRV values highlighted in bold indicate a reliable prediction, as evidenced by the associated mean errors and a narrow standard deviation range of 0.071 < *σ* < 1.30. This signifies minimal deviation in predictions and underscores the reliability of the models. c) Bland–Altman analysis: mean differences (MD) and 95% confidence intervals (MD ± 1.96*σ*) for each ML-based predicted HRV metric compared with the ground truth. The highlighted HRV values are those exhibiting moderate-to-strong agreements between the model predictions and the ground truth with an absolute MD varying according to 0.26 < MD <0.91

a)		
HRV metrics	RMSE	*R*
*Time domain*		
HR (b.p.m.)	**10**.**1**	**0**.**860**
RMSSD (ms)	**4**.**85**	**0**.**581**
SDNN (ms)	40.1 × 10^2^	0.183
TI (—)	**3**.**74**	**0**.**437**
TINN (ms)	106	0.234
*Frequency domain*		
LF (ms^2^)	645	0.253
HF (ms^2^)	**46**.**1**	**0**.**440**
LF/HF (—)	**46**.**7**	**0**.**484**
*Non-linear domain*		
SD1 (ms)	**3**.**66**	**0**.**594**
SD2 (ms)	46.2	0.365
SD1/SD2 (—)	63.6	0.314

b)		
HRV metrics	Mean error	*σ*
*Time domain*		
HR (b.p.m. )	**33**.**7**	**0**.**496**
RMSSD (ms)	**13**.**8**	**0**.**165**
SDNN (ms)	51.5 × 10^2^	48.4
TI (—)	**9**.**01**	**0**.**071**
TINN (ms)	141	0.852
*Frequency domain*		
LF (ms^2^)	671	19.4
HF (ms^2^)	**84**.**2**	**0**.**705**
LF/HF (—)	**93**.**0**	**1**.**29**
*Non-linear domain*		
SD1 (ms)	**9**.**87**	**0**.**117**
SD2 (ms)	92.8	1.123
SD1/SD2 (—)	37.9	0.662

c)		
HRV metrics	MD	(MD ± 1.96*σ*)
*Time domain*		
HR (b.p.m.)	**0**.**908**	**(−19.3; 21.1)**
RMSSD (ms)	**0**.**455**	**(−9.25; 10.2)**
SDNN (ms)	34.0	(−76.8 × 10^2^; 83.6 × 10^2^)
TI (—)	**0**.**590**	**(−6.82; 8.00)**
TINN (ms)	5.01	(−207; 217)
*Frequency domain*		
LF (ms^2^)	−65.0	(−13.6 × 10^2^; 12.3 × 10^2^)
HF (ms^2^)	**−4**.**80**	**(−96.9; 87.3)**
LF/HF (—)	**−4**.**80**	**(−88.5; 98.1)**
*Non-linear domain*		
SD1 (ms)	**−0**.**260**	**(−7.60; 7.08)**
SD2 (ms)	5.19	(−87.1; 97.4)
SD1/SD2 (—)	−10.2	(−136; 116)

Additionally, the model demonstrated strong predictive capabilities for the TI feature, indicative of overall autonomic activity. The Spearman correlation coefficient established a consistent, moderate to strong monotonic relationship (*R* yielded *P* < 0.05 for all), ranging from 0.44 to 0.86 (refer to *Table 5a*). The reliability of our predictions is further affirmed by mean errors and a narrow standard deviation range of 0.071–1.30, underscoring the models’ consistent performance (see *Table 5b*). Furthermore, our models exhibit moderate to strong agreements with the ground truth, as evidenced by an absolute mean deviation ranging from 0.26 to 0.91. These results collectively highlight the acceptable reliability and accuracy of our predictive models for the metrics associated with vagal modulation and the TI feature. The strong agreement found for HRV metrics associated with vagal nerve activity underscores HRV’s robustness as a tool for cardiovascular assessment. This strength is attributed to the well-established link between vagal activity and distinct features within these time series. Notably, significant correlations were evident across various domains: time domain (HR, RMSSD with correlation coefficients of 0.860 and 0.581, respectively), frequency domain (HF and LF/HF with correlation coefficients of 0.440 and 0.484, respectively), and non-linear domain (SD1 with a correlation coefficient of 0.594). It is worth mentioning that while these associations are considered satisfactory, the correlations within the frequency domain appear weaker (*R* < 0.5), indicating somewhat reduced reliability for their practical application.

## Discussion

This is the first feasibility pilot study trial focusing on predicting ANS outcomes quantified through HRV metrics influenced by uncontrolled environmental variables and quantified through GSV image analysis metrics. The findings derived from this study indicate that ML-based models could proficiently anticipate HRV metrics associated with the parasympathetic nerve activity, induced by environmental attributes. Conversely, predictions pertaining to HRV metrics linked to overall (i.e. combined parasympathetic and sympathetic) autonomic activity exhibited weaker agreement with the ground truth. The weaker agreement in metrics reflecting overall autonomic activity, in contrast to those specifically tied to vagal nerve activity like RMSSD, stems from a dearth of distinct physiological correlates. Unlike vagal nerve-related metrics, which benefit from specific physiological markers like HRV during respiratory cycles, metrics encompassing overall autonomic activity lack a comparable level of specificity. The intricate interplay and opposing effects of sympathetic and parasympathetic interactions make it difficult to precisely isolate their individual contributions in composite HRV metrics. Consequently, models predicting overall autonomic activity may experience compromised accuracy, resulting in diminished agreement with ground-truth data.

This pilot study proves the feasibility of using ML-based approaches in potentially predicting HR changes in response to different living environments, which could be related to vagal dynamics, each characterized by unique environmental attributes and features that function as stressors or de-stressors. Once these preliminary results would be improved, effectively capturing HRV responses in the everyday settings of healthy volunteers and patients with CHF during activities marks a notable progress in assessing the ANS in uncontrolled, real-world environments outside the laboratory. This could yield implications of heightened significance, fostering an improved grasp of the ANS’s behaviour within everyday scenarios.

Distinguished from prior research, this study shows the possibility of ML model as a tool to potentially capture psychophysiological reactions prompted by quantified environmental attributes in real-world settings.^11–14,40,41^ These models not only discern patterns across subjects of varying demographics, physical fitness, medical conditions, and activity levels, thereby overcoming limitations posed by controlled settings, but also enable recognition of these patterns. To overcome the challenges associated with uncontrolled settings, our approach explored a different path, concentrating on the objective quantification of ANS responses, reliable indicators of environmental attributes, achieved through HRV measurements at specific GPS coordinates during outdoor activities. Preliminary experiments suggest the significance of vegetation’s presence, as quantified from GSV images, in anticipating HRV parameters indicative of vagal nerve stimulation as they have the highest feature importance in the ML models. These initial findings imply a connection between the extent of vegetation in the living environment and vagal nerve stimulation, thus reinforcing the notion that exposure to natural elements has a quantifiable positive impact on health. Nevertheless, while vegetation’s predominance is noteworthy, it is intriguing that the feature importance of control variables such as activity and age emerges as even more crucial in forecasting HRV parameters related to vagal nerve stimulation, highlighting a potentially more substantial relationship. It is important to note that the pivotal nature of these control variables does not negate the salient role of vegetation. Instead, it highlights the intricate interplay between numerous factors influencing ANS responses, as acknowledged in prior research exploring the connection between natural surroundings, physical activity, and their impact on psychophysiological well-being. Positive outcomes, such as heightened energy levels, have been demonstrated in previous studies.^42^ Similarly, another trial suggests benefits such as improved mood and stress reduction.^43^ Interestingly, the association between human behaviour and environmental characteristics, in terms of both type and size, is worth noting. Notably, natural settings tend to encourage physical activity, with larger natural areas often linked to activities such as walking, jogging, and cycling, all modulating the vagal nerve.^44^

Furthermore, the analysis of feature importance from the ML models unveiled an intriguing finding: the classification of subjects as healthy volunteers or patients diagnosed with CHF did not hold significance in predicting vagal nerve responses. This observation potentially implies that factors such as intensity levels, age, and exposure to natural environments carry equal importance for both population groups in stimulating the vagal nerve, consequently leading to positive health effects. While the current preliminary findings are compelling, their comprehensive extrapolation is restrained by the scope of the dataset. For conclusive insights into the link between vagal nerve stimulation and exposure to natural elements, a broader dataset is vital. This dataset should include more subjects exposed to various environments, each with unique attributes. This will strengthen the validity of the model and our conclusions and enhance our understanding of nature’s impact on well-being.

For practical guidance, it is recommended to harness ML models for predicting HRV metrics, specifically those derived from the time and non-linear domains that mirror parasympathetic nerve responses. These encompass essential parameters such as HR, RMSSD, and SD1. Caution is advised when incorporating metrics from the frequency domain, including HF and LF/HF, due to their lower predictive accuracy and reliability in ML models. In terms of clinical implications, this suggests the application of ML models to support telerehabilitation, with a focus on monitoring changes in HR, RMSSD, and SD1 parameters indicative of parasympathetic nerve responses. Machine learning predictions offer a novel approach to examine and predict HRV responses in both patients with CHF and healthy controls during activities conducted in various environments characterized by diverse environmental attributes. Integrating ML-based data analysis and predictive models into current remote monitoring systems might provide valuable insights for physicians prescribing rehabilitation plans. The ML model could be utilized to identify variations in HRV parameters compared with the patient’s specific baseline. It can categorize these changes as either positive (parasympathetic nerve stimulation) or negative (activating sympathetic overdrive), attributing them to environmental factors and activity intensity. Consequently, physicians might obtain artificial intelligence (AI)-guided insights to recommend personalized interventions, such as GPS walking routes or forest bathing, and identify situations triggering sympathetic overdrive. This approach aims to provide tailored lifestyle advice, enhancing prognosis, and overall patient well-being.

## Limitations and future work

This study has notable limitations, mainly tied to the manual initiation and termination required for collecting continuous HR and GPS signals during outdoor activities, alongside the constraints of a small dataset that restricted the use of advanced techniques such as deep learning. There may also be a mismatch between what the subject was experiencing and what was represented in the GSV images. These factors contribute to the wide limits of agreement in HR estimation, which are currently insufficient for monitoring conditions related to vagal activation.

For future research, it would be recommended to focus on automating data collection with real-time visual representations of the environment, expanding dataset sizes, and implementing feedback systems for personalized health enhancement by categorizing activity types in environments that stimulate the vagal nerve. Pursuing these directions may unveil fresh insights into the intricate relationship between the (urban) living environment, ANS responses, and cardiovascular health.

## Conclusions

Our preliminary results from this pilot study demonstrate that ML algorithms, utilizing data gathered from wearable systems, have the potential to establish a connection between activities conducted in various environments and the vagal nerve response, as it could be reflected in HRV metrics associated with the vagal branch of the ANS. This is evident through the alignment of model predictions with the true ground truth during validation. Consequently, there arises the prospect of employing AI-guided algorithms based on HRV metrics to intelligently process patient data. Such algorithms could guide clinicians in rehabilitation therapy by identifying activities in environments conducive to vagal nerve modulation, particularly in groups exhibiting sympathetic overdrive, like in patients with CHF. Ultimately, this strategy aims to offer tailored telerehabilitation and lifestyle advice, leading to improved prognosis and enhanced overall patient well-being.

## Lead author biography


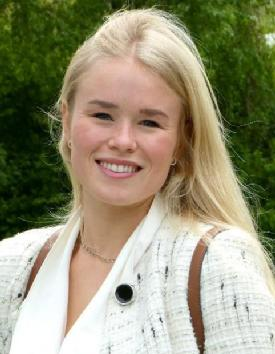


Valerie A.A. van Es holds a dual MSc degree in Medical Engineering-Architecture, Building, and Planning from Eindhoven University of Technology. Currently pursuing a PhD in Cognitive, Computational, and Social Neurosciences at Scuola IMT Alti Studi Lucca, she specializes in ‘Methods for Advanced Biosignal Analyses’. She focuses on developing intelligent algorithms integrated into wearable and nearable technologies for autonomic nervous response monitoring in daily life. Her research aims to create screening tools for a broad population, identifying those at risk of sympathetic overdrive, serving as a proactive warning system to prompt lifestyle changes and prevent cardiovascular diseases.

## Data Availability

The data that support the findings of this study are not publicly available due to privacy and ethical restrictions. Participants did not consent for their data to be shared publicly. As a result, the datasets generated and analysed during the current study are not available.
